# Safe Disposal of Accident Wastewater in Chemical Industrial Parks Using Non-Thermal Plasma with ZnO-Fe_3_O_4_ Composites

**DOI:** 10.3390/toxics12010040

**Published:** 2024-01-04

**Authors:** Aihua Li, Chaofei Wang, Chengjiang Qian, Jinfeng Wen, He Guo

**Affiliations:** 1 College of Safety Science and Engineering, Nanjing Tech University, Nanjing 211816, China; liaihua_njtech@163.com (A.L.);; 2 College of Biology and the Environment, Nanjing Forestry University, Nanjing 210037, China; 19852260029@163.com

**Keywords:** non-thermal plasma, ZnO, ZnO-Fe_3_O_4_, ciprofloxacin, chemical wastewater

## Abstract

Chemical wastewater has a high concentration of toxic and hazardous antibiotic pollutants, which not only devastates the ecological environment and disrupts the ecological balance, but also endangers human health. This research proposed a non-thermal plasma (NTP) combined with a ZnO-Fe_3_O_4_ nano-catalyst system to achieve the efficient degradation of ciprofloxacin (CIP) in chemical wastewater. Firstly, ZnO-Fe_3_O_4_ composite materials were prepared using hydrothermal method and characterized with scanning electron microscopy (SEM), transmission electron microscope (TEM), X-ray diffractometer (XRD), X-ray photoelectron spectroscopy (XPS), etc. With the sole NTP, NTP/ZnO, and NTP/ZnO-Fe_3_O_4_ systems, the removal efficiency of CIP can reach 80.1%, 88.2%, and 99.6%, respectively. The optimal doping amount of Fe_3_O_4_ is 14%. Secondly, the capture agent experiment verified that ·OH, ·O_2_^−^, and ^1^O_2_ all have a certain effect on CIP degradation. Then, liquid chromatography–mass spectrometry (LC-MS) was used to detect the intermediate and speculate its degradation pathway, which mainly included hydroxyl addition, hydroxyl substitution, and piperazine ring destruction. After treatment with the NTP/ZnO-Fe_3_O_4_ system, the overall toxicity of the product was reduced. Finally, a cyclic experiment was conducted, and it was found that the prepared ZnO-Fe_3_O_4_ catalyst has good reusability. The NTP/ZnO-Fe_3_O_4_ was also applied in practical pharmaceutical wastewater treatment and has practical applicability.

## 1. Introduction

Chemical industrial parks are the concentration areas of modern chemical industries, playing an important role in promoting economic development. However, due to the frequent involvement of toxic and harmful substances in the production process of chemical parks, especially antibiotic pollutants, once accidents occur, they may cause severe environmental pollution and personnel harm [1,2,3]. Therefore, it is very important to establish a comprehensive emergency response plan for pollution accidents in chemical industrial parks, which can in a timely manner and effectively respond to accidents and minimize losses.

Advanced oxidation method can generate strong oxidizing free radicals (such as hydroxyl radical (·OH)), which can completely degrade organic matter into CO_2_ and H_2_O [4]. The commonly used advanced oxidation methods currently include Fenton method, photocatalytic method, electrochemical method, and ozone catalytic oxidation method [5]. However, these methods generally have associated problems such as harsh reaction conditions, complex operations, and secondary pollution [6]. Non-thermal plasma (NTP) is an emerging advanced oxidation technology in recent years [7]. During the discharge process under applied voltage, it dissociates gas phase molecules and water molecules, producing a large number of active free radicals. It also radiates physical effects such as ultraviolet light and shock waves. Due to its advantages of being fast and efficient, causing no secondary pollution, and having a simple operation, NTP has been proposed for the degradation of organic pollutants in water, mainly including dyes, persistent organic pollutants, endocrine disruptors, and antibiotics [8]. In recent decades, research on plasma technology has also been very active, providing new technologies, methods, and processes for synthesizing new substances and materials and for environmental pollution control. However, its low energy utilization has always limited its development due to the insufficient utilization of its optical and chemical effects [9]. Therefore, it is necessary to seek suitable catalysts to combine with NTP for improving the energy utilization of NTP and the removal efficiency of organic pollutants.

ZnO has attracted widespread attention from researchers due to its stable physical and chemical properties, non-toxicity, rich morphology, and low cost [10]. Previous studies have reported that NTP can successfully activate ZnO for photocatalytic reactions, thereby improving the efficiency of plasma energy utilization and organic matter removal [11,12,13,14]. However, ZnO is an n-type semiconductor material with a wide band gap, which limits its ability to utilize near-ultraviolet light with shorter wavelengths and higher energy. It is worth noting that NTP generates a wide range of light, including both ultraviolet and visible light [15]. On the other hand, the generated electron–hole pairs in ZnO are more prone to recombination, resulting in low actual utilization efficiency of photons and insufficient photocatalytic activity. In addition, ZnO nanoparticles are difficult to recover in NTP water treatment systems, which are not conducive for secondary use.

Based on the above issues, some studies consider loading Fe_3_O_4_ onto ZnO nanoparticles [16]. The ZnO-Fe_3_O_4_ nanocomposite material can not only shorten the band gap and improve the light absorption range but also reduce the recombination of electrons and holes and improve the photocatalytic performance [17]. In addition, ZnO-Fe_3_O_4_ nanocomposites also have magnetism, which facilitates separation, recycling, and reuse [18]. However, this material requires an external light source, and the long processing time results in insufficient photocatalytic degradation efficiency. Therefore, this study considers constructing an NTP/ZnO-Fe_3_O_4_ system as a UV-stimulated luminescent catalyst for NTP radiation, which not only enhances its photocatalytic activity but also enhances the energy utilization efficiency of NTP, achieving the goal of short-term and efficient degradation of pollutants. However, as far as we know, there is still a significant gap in the research on NTP assisted ZnO-Fe_3_O_4_ nanocomposites for wastewater treatment, and the related degradation pathways and mechanisms are not yet thoroughly studied.

This research proposed the NTP/ZnO-Fe_3_O_4_ nano-catalyst system to achieve the efficient degradation of ciprofloxacin (CIP) in chemical wastewater. Firstly, ZnO-Fe_3_O_4_ nanocomposites were synthesized using hydrothermal method and characterized with scanning electron microscopy (SEM), transmission electron microscope (TEM), X-ray diffractometer (XRD), and X-ray photoelectron spectroscopy (XPS). Secondly, this study verified the effect of composite catalyst ZnO-Fe_3_O_4_ and investigated the effects of different doping and addition amounts of composite catalyst on the degradation efficiency of CIP. Then, the generation and action of free radicals were analyzed using trapping agent experiments. Based on the results of three-dimensional fluorescence, liquid chromatography–mass spectrometry (LC-MS) and DFT analysis decipher the degradation pathway of CIP and determine the catalytic and degradation mechanisms. In addition, to verify whether this system can reduce the toxicity of pollutants, this study used the typical toxicity analysis software ECOSAR (Version 2.0) to analyze the toxicity of CIP and its intermediate products. Finally, this study also conducted catalyst recovery and cycling performance tests and verified whether the NTP/ZnO-Fe_3_O_4_ system can be applied to the treatment of typical pharmaceutical wastewater. This study constructs the NTP/ZnO-Fe_3_O_4_ system and delves into the corresponding reaction mechanism, hoping to fill the gap in its application in wastewater treatment and provide some reference for subsequent research.

## 2. Experimental

### 2.1. Reagents

Zinc acetate, sodium hydroxide (NaOH), hydrogen peroxide, thick sulfuric acid, ethylene glycol, iron chloride, and ferrous sulfate were bought from China National Pharmaceutical Group Chemical Reagent Co., Ltd. (Beijing, China) CIP, isopropyl alcohol, benzoquinone, sodium oxalate, ethanolamine, and sodium acetate were purchased from Aladdin Biotechnology Co., Ltd (Shanghai, China).

### 2.2. Experimental Setup and Analysis

**Figure 1:**
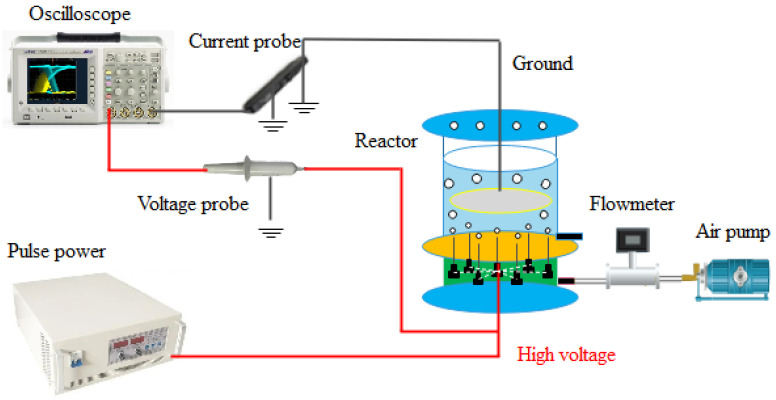
Experimental system diagram.

The experimental system is shown in Figure 1 and mainly includes a power supply, an oscilloscope, and a reactor. The driving power supply is a high-voltage pulse power supply. The oscilloscope (Tektronix, Beaverton, OR, USA, MDO3012) was used to detect the voltage (Tektronix, USA, MDO3012) and current characteristics (Tektronix, USA, P6021) during the discharge process. It was equipped with voltage and current probes, and the voltage and current waveform during the discharge process were displayed in real time on the oscilloscope. The reactor was composed of needle plate electrodes, and the gas flowed through the flow meter through a gas pump to a drum into the reactor, ultimately forming a bubble plasma. During the experiment, the prepared catalyst was placed in the reactor, and the physicochemical effects generated by plasma discharge can activate the catalyst to further undergo catalytic reactions, thereby improving the pollutant removal efficiency. During the experiment, samples were taken every five minutes using a syringe. The concentration of CIP was detected using a high-performance liquid chromatograph (HPLC). This chromatographic method was equipped with a symmetrical C18 column (Elite, 4.6 mm × 250 mm). The detection wavelength was 276 nm. The mobile phase consisted of acetonitrile and 0.01 M phosphoric acid (pH 2.5) in a ratio of 20:80 *v*/*v*, with a flow rate of 0.6 mL/min. The injection volume and residence time were 20 mL and 15 min, respectively.

### 2.3. ZnO-Fe_3_O_4_ Preparation and Characterization

ZnO was prepared using hydrothermal method. Firstly, 1 g of zinc acetate was dissolved in 120 mL of ethylene glycol solution, which was then stirred in a 70 °C water bath for 1 h. Then, the mixture solution was dispersed using ultrasonic treatment for 1 h, and NaOH solution was then added to the above solution. Then, it was stirred at 70 °C for 2 h. The solution was placed in a polytetrafluoroethylene reactor and reacted at 160 °C for about 20 h. Finally, the solid particles were centrifuge washed with deionized water and anhydrous ethanol. The obtained ZnO nanoparticles were dried in a vacuum drying furnace (70 °C) for 10 h.

The prepared ZnO was dissolved in 30 mL ethylene glycol solution and was added to 683 mg iron chloride and 584 mg ferrous sulfate. Adjusting the doping amount of Fe_3_O_4_ can be achieved by proportionally changing the amounts of iron chloride and ferrous sulfate. Then, 0.5 g sodium acetate was added to the suspension after stirring. This solution was further stirred for 0.5 h at 80 °C. To completely mix the colloidal particles, 15 mL ethanolamine was added to the suspension, then heated at 150 °C for 6 h. Finally, the solution was cooled at room temperature and washed with deionized water. The obtained ZnO-Fe_3_O_4_ composites were dried at 60 °C for 12 h. In the studies, the prepared ratio of ZnO to Fe_3_O_4_ was 7%, 14%, and 21%.

The morphologies of ZnO-Fe_3_O_4_ samples were characterized using SEM (FEI, Nova Nano, Lincoln, NE, USA, SEM 450) and TEM (Tecnai F30, FEI, Hillsboro, OR, USA). The structure was tested via XRD (Bruker D8 Focus, Ettlingen, Germany). XPS (Thermo, Waltham, MA, USA, ESCALAB 250X) was adopted for the chemical state of various elements on the sample surface. FT-IR spectra were performed on an FT-IR spectrometer (Bruker MPA FTIR). The ultraviolet–visible diffuse reflectance spectrum (UV-Vis DRS) was determined using a ultraviolet–visible spectrophotometer (Shimadzu, Kyoto, Japan, UV-550) with barium sulfate as the reference material.

### 2.4. Toxicity Analysis Using ECOSAR

In the toxicity test, the aquatic toxicity of a substance can be predicted according to its structural similarity. To predict the acute and chronic toxicity of CIP and its degradation intermediates, the ECOSAR tool was used [19]. In the calculation process, fish, Daphnia, and green algae were selected as targets.

## 3. Results and Discussion

### 3.1. Characterization

**Figure 2:**
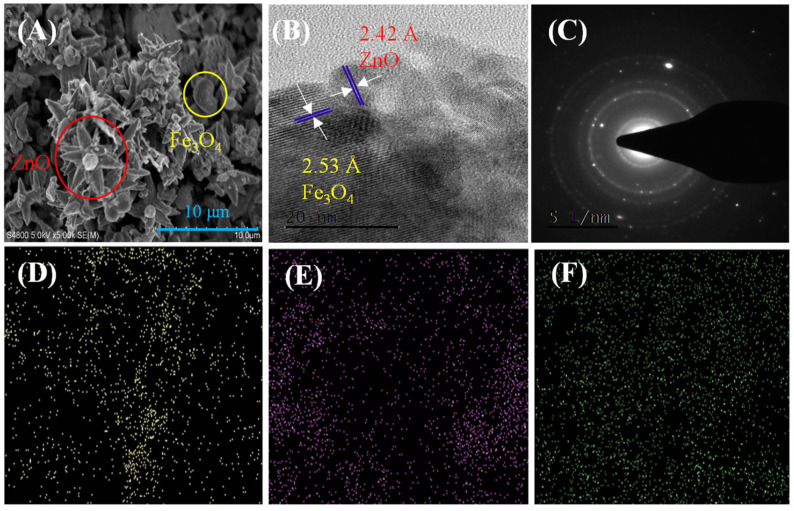
(**A**) SEM, (**B**) TEM, and (**C**) SAED patterns of ZnO-Fe_3_O_4_; EDS mapping: (**D**) Zn, (**E**) Fe, and (**F**) O.

The morphology of ZnO-Fe_3_O_4_ composite material was characterized using SEM and TEM, and the results are shown in Figure 2. From Figure 2A, it can be seen that ZnO exhibits a nanoflower-like structure, while Fe_3_O_4_ exhibits a granular spherical structure, with direct overlap between the two. Figure 2B shows a high-power scanning electron microscope image, where the lattice spacing of 2.42 Å and 2.53 Å belongs to ZnO and Fe_3_O_4_ [14,20], respectively. In addition, there is also a phenomenon of interlacing between their lattices. Figure 2C shows the diffraction pattern of ZnO-Fe_3_O_4_, with its circular structure belonging to the crystal planes of ZnO and Fe_3_O_4_, indicating that the material is indeed composed of ZnO and Fe_3_O_4_ composites. In particular, the bright spot may come from the interference of other elements of the testing instrument.

EDS analysis was performed on the prepared composite materials. Figure 2D–F show the distribution of Zn, Fe, and O elements, respectively. It can be seen that Zn, Fe, and O are evenly distributed on the surface of ZnO-Fe_3_O_4_ composites, and the content of Fe and O is higher than that of Zn, which is also consistent with the SEM image of the composite. In summary, ZnO-Fe_3_O_4_ composite catalyst samples can be successfully prepared.

**Figure 3:**
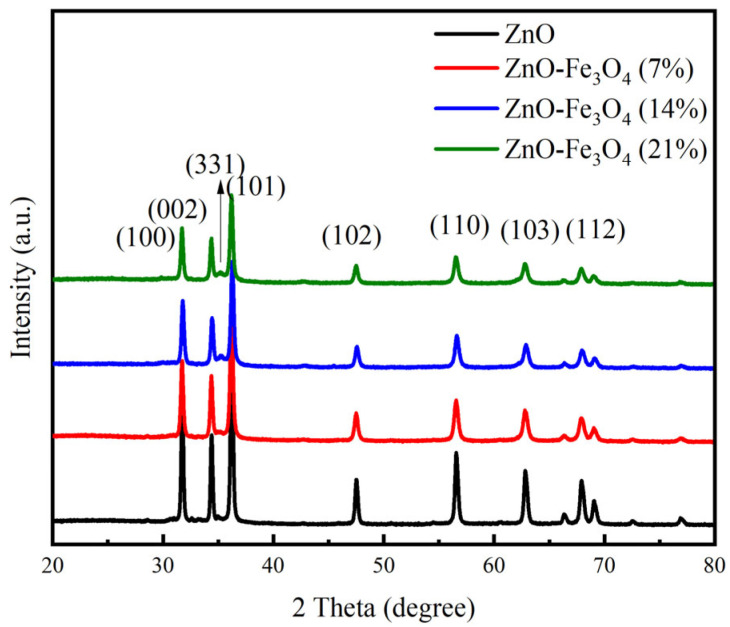
XRD patterns of ZnO-Fe_3_O_4_ composites.

**Figure 4:**
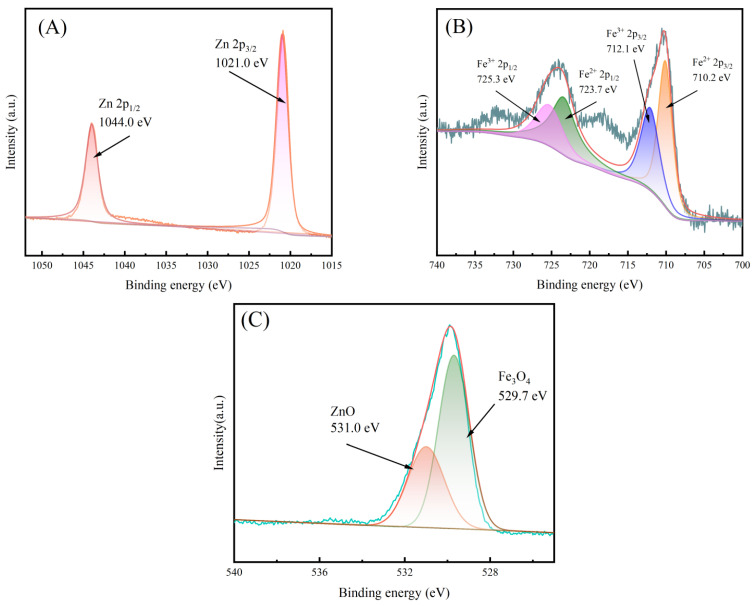
XPS spectra of (**A**) Zn, (**B**) Fe, (**C**) O.

XRD was used to detect the crystal phases of ZnO-Fe_3_O_4_ composite materials with different doping levels. Figure 3 shows two sets of diffraction peaks of ZnO-Fe_3_O_4_ composite material. One group is the diffraction peaks of ZnO, (100), (002), (101), (102), (110), (103), and (112) crystal planes [21], corresponding to 2θ = 31.7°, 34.4°, 36.2°, 47.5°, 56.5°, 62.8°, and 67.8°, respectively. The other group is the diffraction peak of Fe_3_O_4_, the (331) crystal plane, which corresponds to 2θ = 35.2°. From Figure 3, it can be seen that the XRD patterns of ZnO-Fe_3_O_4_ all show the 35.2° diffraction peak belonging to the (331) crystal plane of Fe_3_O_4_ [20], indicating that the material was successfully synthesized from ZnO and Fe_3_O_4_. Figure 4 shows the XPS spectra of Zn, Fe, and O elements. The peaks at 1021.0 eV and 1044.0 eV represent Zn 2p3/2 and Zn 2p1/2, respectively (Figure 4A) [22]. Fe^2+^2p3/2, Fe^3+^2p3/2, Fe^2+^2p1/2, and Fe^3+^2p1/2 have four peaks at 710.2 eV, 712.1 eV, 723.7 eV, and 725.3 eV, respectively (Figure 4B) [23]. The peak of O element is composed of ZnO and Fe_3_O_4_, and there is an overlap phenomenon between the two peaks (Figure 4C), indicating that a phase separation has occurred [24].

### 3.2. Catalytic Degradation Performance of ZnO-Fe_3_O_4_ Composites

**Figure 5:**
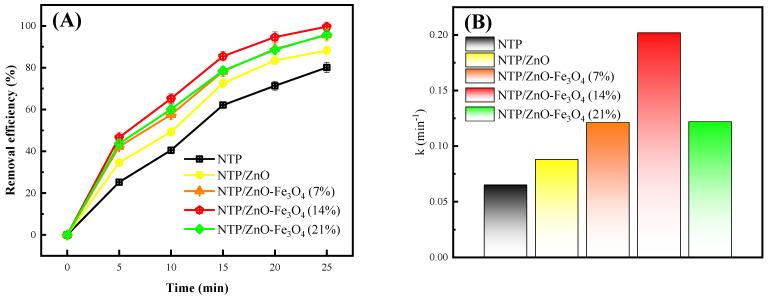
Effect of Fe_3_O_4_ doping amount: (**A**) performance and (**B**) kinetic constant. Experimental conditions: peak voltage of 19 kV; air flow rate of 4 L/min; electrode gap of 10 mm; and catalyst dosage of 0.28 g/L.

The catalytic effects of different catalysts in NTP system were investigated, and the results were shown in Figure 5. It can be seen that the addition of catalysts can improve the removal efficiency of CIP compared to a separate NTP system. The addition of ZnO alone can increase the removal efficiency of CIP from 80.1% to 88.2% (as Figure 5A). The addition of ZnO-Fe_3_O_4_ can further improve the removal efficiency of CIP. With the increase in Fe_3_O_4_ doping amount, the removal first increased and then decreased. When the doping amounts were 7%, 14%, and 21%, the removal rates reached 95.6%, 99.6%, and 95.9%, respectively. Obviously, when the Fe_3_O_4_ doping amount was 14%, the removal efficiency of CIP reached its highest. Figure 5B shows the effect of different catalysts on the first-order kinetic constant. It can be seen that the addition of ZnO can enhance the kinetic constant. The doping of Fe_3_O_4_ can further enhance the kinetic constant, and a 14% doping amount achieves the maximum kinetic constant. In the NTP, NTP/ZnO, and NTP/ZnO-Fe_3_O_4_ (14%) systems alone, the kinetic constants can reach 0.065 min^−1^, 0.088 min^−1^, and 0.202 min^−1^, respectively.

In the NTP system, the light and electronic effects generated by discharge can stimulate the photocatalytic reaction of ZnO (Equations (1)–(4)), thereby improving the removal efficiency of CIP. As for the individual ZnO system, the doping of Fe_3_O_4_ can shorten the band gap of ZnO and enhance its range corresponding to visible light [25]. At the same time, Fe_3_O_4_ can capture the conduction band electrons of ZnO, suppress the recombination of electron holes, and improve photocatalytic performance [26]. In addition, it is worth noting that Fe_3_O_4_ itself can also undergo heterogeneous Fenton reactions in the plasma system, catalyzing H_2_O_2_ to produce more ·OH (Equation (5)) [20]. However, excessive Fe_3_O_4_ doping actually makes it a composite center for electrons and holes of ZnO, thereby reducing the photocatalytic effect [27]. In the end, the removal efficiency of CIP decreases.

plasma + ZnO → e^−^ + h^+^ (1)

O_2_ + e^−^ → O_2_·^−^ (2)

h^+^ + H_2_O → H^+^ + ·OH (3)

h^+^ + OH^−^ →·OH (4)

Fe^2+^_surf._ + H_2_O_2_ → Fe^3+^_surf._+ OH^−^ +·OH (5)

**Figure 6:**
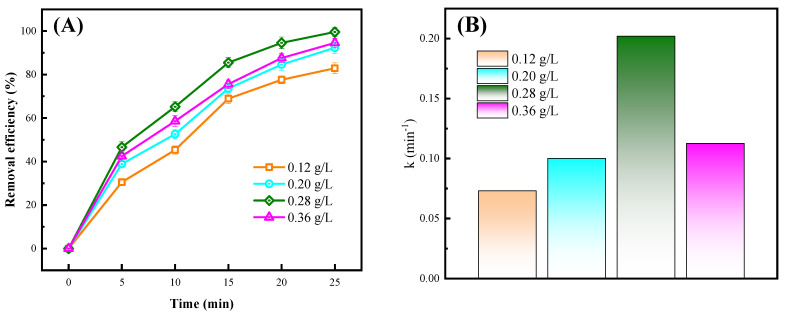
Effect of ZnO-Fe_3_O_4_ dosage: (**A**) performance and (**B**) kinetic constant. Experimental conditions: peak voltage of 19 kV; air flow rate of 4 L/min; and electrode gap of 10 mm.

The addition amounts of different catalysts are shown in Figure 6. It can be seen that as the addition amount increased, the removal efficiency first increased and then decreased. When the addition amount was 0.28 g/L, the removal efficiency reached its optimal value, which was 99.6%. Similarly, the kinetic constants also increased first and then decreased. When the addition amount was 0.12 g/L, 0.20 g/L, 0.28 g/L, and 0.36 g/L, the kinetic constants reached 0.073 min^−1^, 0.100 min^−1^, 0.202 min^−1^, and 0.113 min^−1^, respectively. As the addition amount increased, the catalytic effect became more significant. However, when the addition amount reached a certain value, the catalysts covered each other, and the effective contact area decreased. In addition, when too much catalyst was present, the transparency of aqueous solution decreased, resulting in low light energy utilization [28]. Therefore, an optimal amount of catalyst added, as a higher amount of catalyst was not necessarily better for the reaction.

### 3.3. Role of Active Species

**Figure 7:**
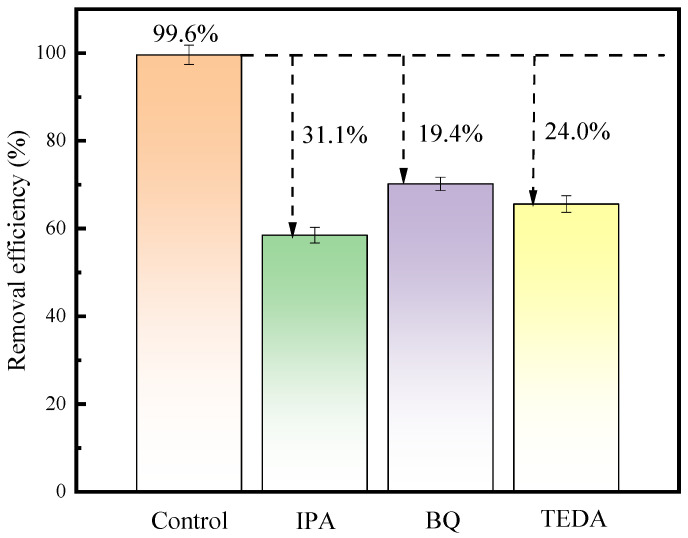
Role of various active species. Experimental conditions: peak voltage of 19 kV; air flow rate of 4 L/min; electrode gap of 10 mm; and catalyst dosage of 0.28 g/L.

To clarify the role of free radicals, capture agent experiments were conducted. IPA, BQ, and TEDA were selected to capture ·OH, ·O_2_^−^, and ^1^O_2_, respectively [29]. As shown in Figure 7, it can be seen that the addition of free radicals leads to a decrease in removal efficiency. The addition of IPA reduced the removal rate of CIP from 99.6% to 68.5%. When BQ was present in the solution, the CIP removal rate decreased from 99% to 80.2%. Similarly, TEDA reduced the CIP removal rate from 99.6% to 75.6%. The addition of IPA, BQ, and TEDA reduced the removal rate of CIP by 31.1%, 19.4%, and 24.0%, respectively, indicating that ·OH, ·O_2_^−^, and ^1^O_2_ all played a certain role.

### 3.4. Degradation Process and Toxicity Variation

**Figure 8:**
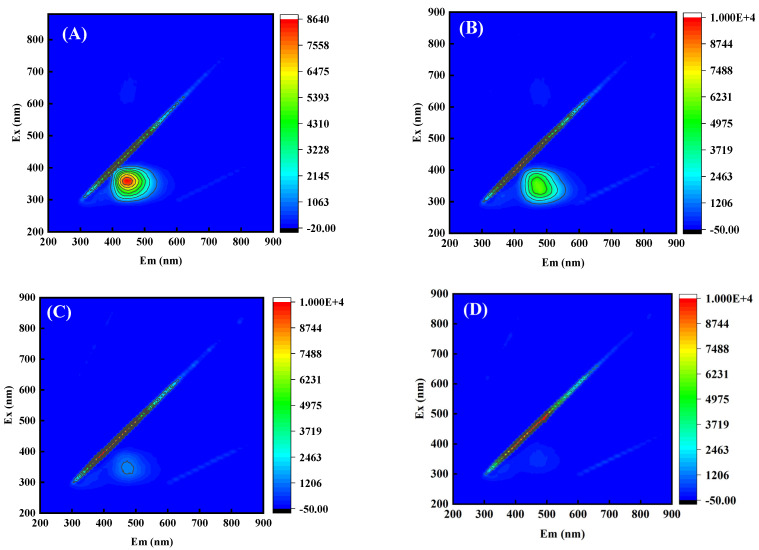
Three-dimensional fluorescence spectra of CIP solution after different treatment times: (**A**) 0 min; (**B**) 10 min; (**C**) 20 min; and (**D**) 30 min.

The degradation process of CIP was thoroughly identified using three-dimensional fluorescence spectrometry, and the results are shown in Figure 8. There was one main peak in CIP solution, which was located at Ex/Em 400–500/300–400 nm in untreated CIP solution (Figure 8A), indicating that CIP molecules had the structural properties of humic acid [30]. With the progress of the reaction, the peak obviously decreased, indicating that its corresponding structure was destroyed (Figure 8B,C). After 30 min of treatment, the original main peaks of CIP basically disappeared (Figure 8D), indicating that CIP molecules can be decomposed completely.

**Figure 9:**
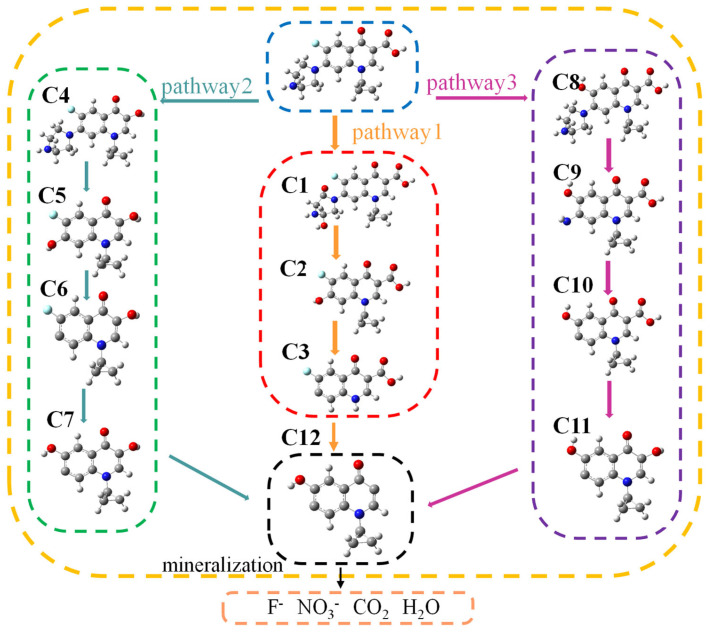
Proposed degradation pathway.

In order to clarify the degradation pathway of CIP, we analyzed the degradation products. There were 12 intermediate products, which were detected through LC-MS analysis. Based on the identified intermediates, three specific degradation pathways were analyzed and are depicted in Figure 9, mainly involving processes such as hydroxyl addition, hydroxyl substitution, and piperazinyl ring destruction. Pathway 1: Hydroxyl groups first attacked the piperazine ring of CIP to generate C1 [31]. The hydroxyl group further attacked the C-N bond between the piperazine ring and the benzene ring, causing the piperazine ring to detach and generate C2. The cyclopropyl fragment from the quinolone moiety on C2 was further attacked by free radicals, resulting in its detachment and the generation of C3. C3 was transformed into C12 through decarboxylation. Pathway 2: CIP firstly undergoes decarboxylation reaction to generate C4 [32], which is then converted into C5 through the oxidation of piperazine by hydroxyl radicals. The hydroxyl groups on the benzene ring of C6 detached to form C7, and its F atom was replaced by hydroxyl groups to form C8. C8 was further converted into C12 through hydroxyl shedding. Pathway 3: The F atom on the CIP benzene ring was first attacked by hydroxyl groups and underwent a substitution process to form C8 [33]. The piperazine ring of C8 was further attacked by free radicals, causing its C-N bond to break and generate C9. The residual C-N bond of C9 piperazine ring was further attacked by free radicals, causing it to detach and generate C10. The carboxyl group on C10 was further replaced by hydroxyl groups to form C11, which was then removed and ultimately formed C12. These intermediate products were finally mineralized, resulting in the production of F^−^, NO_3_^−^, CO_2_, and H_2_O [34,35].

**Figure 10:**
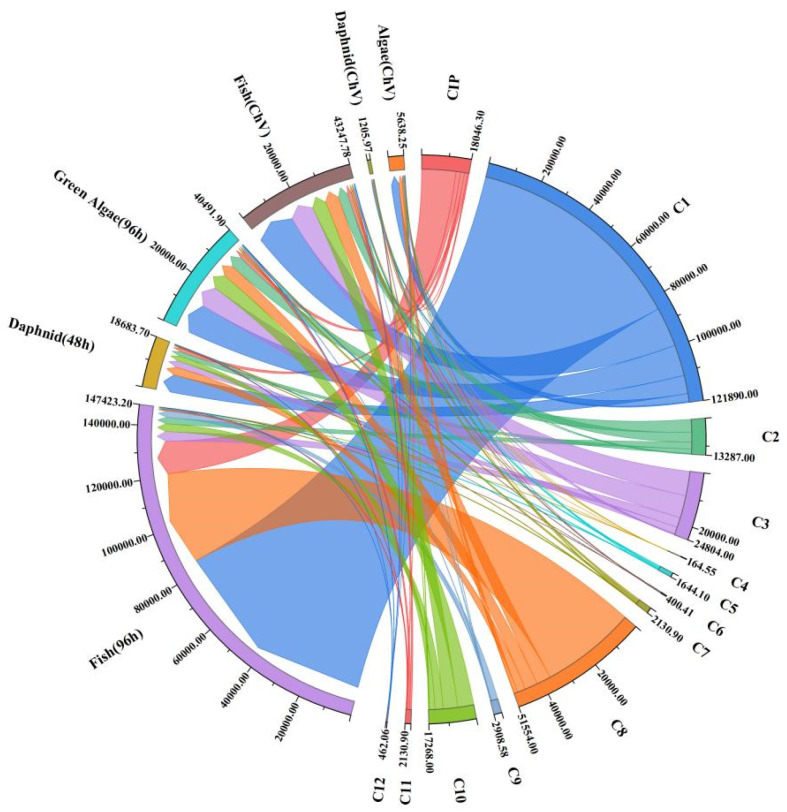
Toxicity of degradation intermediates.

In addition, the toxicities of intermediates were analyzed using ECOSAR software, and the data are depicted in Figure 10. The toxicity of the corresponding substance is represented by the thickness of the string plot. From Figure 10, it can be seen that the toxicities of C1, C2, and C3 in pathway 1 are lower than that of CIP. The toxicities of C4 and C6 in pathway 2 are slightly higher, but the overall toxicity still decreases. The toxicity in pathway 3 is also significantly reduced, especially in product C8. Overall, most of the intermediate products in the NTP/ZnO-Fe_3_O_4_ system for CIP treatment did not exhibit a high toxicity, and their toxicities were far below international standards. Therefore, treatment with the NTP/ZnO-Fe_3_O_4_ system can reduce the toxicities of pollutants.

**Figure 11:**
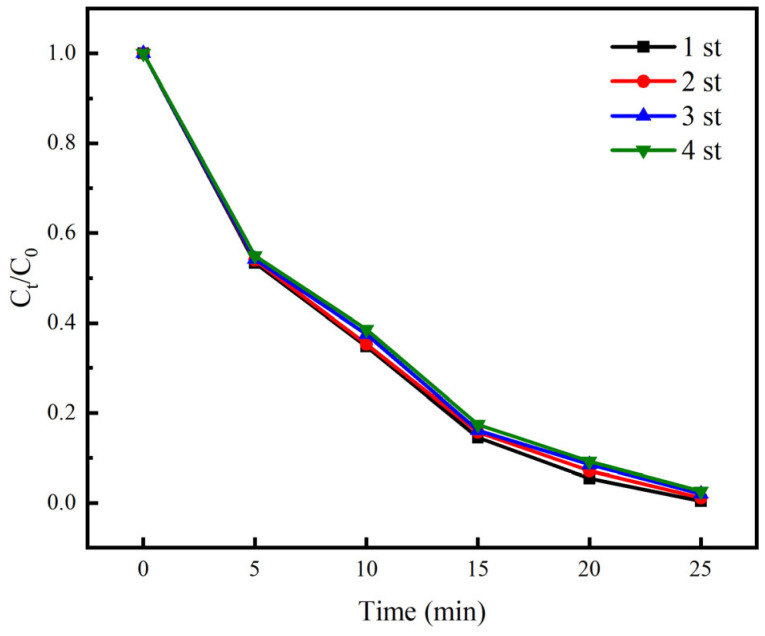
Recycle performance of ZnO-Fe_3_O_4_.

In addition to its superior photocatalytic performance, the magnetic properties facilitate recycling, which is also one of the significant advantages of ZnO-Fe_3_O_4_ composite materials. In order to investigate the cycling performance of ZnO-Fe_3_O_4_ composite catalyst, multiple magnetic recovery cycling tests were conducted on the catalyst. The results are shown in Figure 11. From Figure 11, it can be seen that after four experiments, the removal rate of CIP decreased from the original 99.6% to 97.4%, indicating that the catalytic performance of ZnO-Fe_3_O_4_ composite material has hardly decreased. This indicates that the ZnO-Fe_3_O_4_ composite nanomaterials prepared in this study have good stability and can be reused in the NTP/ZnO-Fe_3_O_4_ system while maintaining a high pollutant degradation rate.

### 3.5. Reuse Performance and Actual Wastewater Treatment

**Figure 12:**
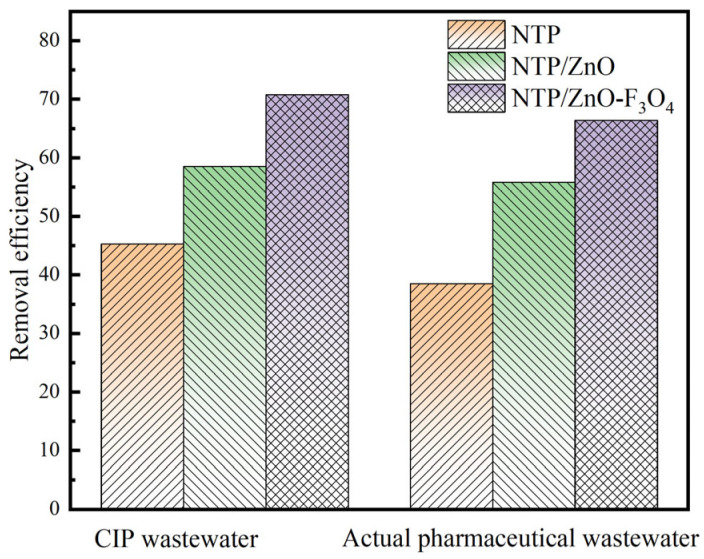
COD removal of CIP wastewater and actual pharmaceutical wastewater.

The actual water quality situation is complex and variable, often containing various organic pollutants that are difficult to degrade. Most wastewater treatment methods have the problem of low mineralization rate and poor biodegradability of treated wastewater. In order to investigate the biodegradability of pollutant solutions treated with the NTP/ZnO-Fe_3_O_4_ system, CIP wastewater and actual pharmaceutical wastewater were selected as target pollutants, and their chemical oxygen demand (COD) after reaction was measured and plotted as shown in the bar graph in Figure 12. As shown in Figure 12, the addition of ZnO in the NTP system increases the COD removal rates of CIP wastewater and pharmaceutical wastewater by approximately 13.0% and 14.0%, respectively. The addition of ZnO-Fe_3_O_4_ in the NTP system increases the COD removal rates of CIP wastewater and pharmaceutical wastewater by approximately 27.0% and 19.0%, respectively. Overall, the COD removal efficiency of the NTP/ZnO-Fe_3_O_4_ system for wastewater is around 70.0%, indicating that the system can effectively degrade pollutants, improve the biodegradability of wastewater solutions, and has good practical application value.

## 4. Conclusions

In this study, an emergency response scheme for the treatment of chemical wastewater by NTP/ZnO-Fe_3_O_4_ system was established with CIP as the target pollutant. Firstly, ZnO-Fe_3_O_4_ composite materials were successfully prepared using hydrothermal method and characterized with SEM, TEM, XRD, XPS, etc. Secondly, the experiment found that the removal rate of CIP with the NTP/ZnO-Fe_3_O_4_ system (99.6%) increased by 19.5% and 11.4% compared to that of the single NTP and NTP/ZnO systems, respectively. The optimal doping amount of ZnO-Fe_3_O_4_ is 14%, and the optimal dosage is 0.28 g/L. Secondly, the capture agent experiment found that active substances such as ·OH, ·O_2_^−^, and ^1^O_2_ all play an important role in the degradation of CIP. Then, the specific degradation pathways of CIP were proposed through three-dimensional fluorescence spectroscopy and LC-MS analysis, including hydroxyl addition, hydroxyl substitution, and piperazine ring disruption. In addition, after treatment with the NTP/ZnO-Fe_3_O_4_ system, the overall toxicity of the product is reduced. Finally, cyclic experiments and actual wastewater treatment experiments have shown that ZnO-Fe_3_O_4_ can maintain a high catalytic effect even after repeated use, and the NTP/ZnO-Fe_3_O_4_ system can improve the biodegradability of wastewater and is suitable for various practical wastewater treatments. In summary, the NTP/ZnO-Fe_3_O_4_ system not only provides new ideas for the green and efficient treatment of chemical wastewater but also further enriches the knowledge of the relevant reaction mechanisms, providing reference for subsequent research by scholars.

## Data Availability

The data presented in this study are available on request from the corresponding author.
